# Plastic lengthening amputation with vascularized bone grafts in children with bone sarcoma: a preliminary report

**DOI:** 10.1186/s12957-020-02020-5

**Published:** 2020-09-15

**Authors:** Zhiqiang Zhao, Qinglin Jin, Xianbiao Xie, Yongqian Wang, Tiao Lin, Junqiang Yin, Gang Huang, Changye Zou, Jingnan Shen

**Affiliations:** 1 Department of Musculoskeletal Oncology, The First Affiliated Hospital of Sun Yat-Sen University, Guangzhou, China; 2 Guangdong Provincial Key Laboratory of Orthopedics and Traumatology, Guangzhou, 510080 China

**Keywords:** Sarcoma, Limb, Amputation, Resection, Reconstruction

## Abstract

**Background:**

At present, amputation was widely adopted for young patients when limb salvage was deemed risky with several surgical strategy such as rotationplasty. However, leg length discrepancies and unfavorable cosmetic results were indispensable complication of this strategy. The purpose of this study was to propose a novel reconstruction strategy and evaluate the early clinical and functional outcomes of the strategy.

**Methods:**

Plastic lengthening amputation (PLA) has been developed by lengthening the stump to preserve one additional distal joint for fixing the artificial limb well. The surgical technique and postoperative management were documented, and the functional outcomes were compared with those of traditional amputation (TA). Six pairs of patients matched for age, sex, location, pathological type, and final prosthesis underwent individually designed plastic lengthening amputation with vascularized autografts or traditional amputation between January 2005 and December 2007. All patients were followed, and the locomotor index and the musculoskeletal tumor society score (MSTS) were used to describe and quantitatively grade limb functional outcomes after amputation. The complications and functional outcomes of the patients taken two kinds of procedures were compared.

**Results:**

Twelve patients with osteosarcoma or Ewing’s sarcoma of either the femur or tibia were included in the study. Six patients underwent plastic lengthening amputations, three of whom also underwent vascular anastomosis. Patients were followed for an average of 48.17 months; bone healing required an average of 3.3 months. No local recurrence was found. The average postoperative locomotor index functional score of the affected limb was 32.67 ± 5.89 in the plastic lengthening amputation group while was 19.50 ± 7.87 in the traditional amputation group. The MSTS functional scores were 22.67 ± 1.33 and 24.17 ± 1.45 at 6 and 12 months for patients in PLA group while 17.00 ± 1.549 and 17.83 ± 1.64 at 6 and 12 months for patients in TA group.

**Conclusions:**

Plastic lengthening amputations with vascularized autografts could preserve the knee joint to improve the function of the amputated limb in selected bone sarcoma patients.

## Background

At present, with the advancements in diagnosis, chemotherapy, and surgical techniques, limb salvage surgery with increased long-term survival rates has been widely conducted for primary malignant bone tumors [1, 2]. However, limb-salvaging surgery remains challenging to musculoskeletal oncologists due to several difficulties including unplanned surgery, partial main vascular involvement, inadequate amounts of soft tissue covering, high postoperative expenses, and leg length discrepancies [3, 4]. Therefore, amputation is adopted for young patients when limb salvage is deemed too risky. Rotationplasty has been frequently used as a functional reconstruction method in young patients who suffer from knee joint tumors [5, 6]. Although the ankle joint partly compensates for knee joint function and improves the function of the residual limb, most patients and their parents have opposed this surgery due to leg length discrepancies and unfavorable cosmetic results [7, 8].

We have been performing the newly developed plastic lengthening amputation with vascularized bone grafts and proximal epiphysis preservation since 2005; this procedure creatively transforms above-knee amputation to below-knee amputation or transforms hip disarticulation into above-knee amputation in order to improve the function of the affected limb. In this retrospective cohort study, we compared a plastic lengthening amputation group with a traditional amputation group.

## Methods

### Ethical statement

This study was approved by the Ethical Committee of the First Affiliated Hospital of Sun Yat-sen University with the reference number of [2008]-No. 9. Plastic lengthening amputation with vascularized autografts has been performed since January 2005 in the First Affiliated Hospital of Sun Yat-sen University; this procedure was approved by the Sun Yat-sen University Ethics Committee and has therefore been performed in accordance with the ethical standards put forth in the 1964 Declaration of Helsinki. Consent from patients or their guardians was obtained before patient inclusion in the study.

### Study design and population

A retrospective cohort study was undertaken in six pairs of patients with the same age, sex, location, and pathologic type underwent plastic lengthening amputation (PLA) or traditional amputation (TA) at the First Affiliated Hospital of Sun Yat-sen University for the treatment of sarcoma of the femur or tibia from January 2005 to December 2007. The patients with unresectable tumors in the lower limb were undertaken PLA or TA according to the choice of patients. Four patients were male, and eight patients were female. The average age was 9 years old (range 6 to 13 years old). All patients suffered from pain and had a palpable mass at presentation. Core needle biopsies were performed in four patients, six patients underwent contaminated unplanned surgery, and two patients had undergone an open biopsy in other hospitals. There were 10 patients with osteosarcoma and 2 with Ewing’s sarcoma. Four of those patients had been given surgical treatment in local hospitals with misdiagnosis. All the patients had Enneking stage IIB disease [9]. The patients’ information is summarized in Table 1.

**Table 1:** The 6 pairs of patients were chosen with the same age, sex, location, pathologic type, and the final outcome

Patients pair no.	Location	Pathologic type	Stage	Procedure	Vascular anastomosis	Amputation Level	Bone union	The locomotor index	Follow-up (month)	Outcome	MSTS functional score (6 months-12 months)	
1	Tibia	Osteosarcoma	IIB	PLA	No	TT	3	39	76	AWD	25	28
1	Tibia	Osteosarcoma	IIB	TA	No	TF	No	25	71	AWD	20	21
2	Femur	Osteosarcoma	IIB	PLA	Yes	TF	4	24	25	DOD	19	21
2	Femur	Osteosarcoma	IIB	TA	No	DH	No	10	62	AWD	14	15
3	Tibia	Osteosarcoma	IIB	PLA	No	TT	3	34	63	AWD	22	24
3	Tibia	Osteosarcoma	IIB	TA	No	TF	No	22	28	DOD	12	13
4	Tibia	Osteosarcoma	IIB	PLA	No	TT	2.5	37	52	AWD	27	27
4	Tibia	Osteosarcoma	IIB	TA	No	TF	No	25	49	AWD	21	23
5	Tibia	Osteosarcoma	IIB	PLA	Yes	TT	3	35	46	AWD	24	26
5	Tibia	Osteosarcoma	IIB	TA	No	TF	No	26	32	DOD	20	20
6	Femur	Ewing’s sarcoma	IIB	PLA	Yes	TF	4.5	27	38	AWD	19	21
6	Femur	Ewing’s sarcoma	IIB	TA	No	DH	No	9	36	AWD	15	15

*PLA* plastic lengthening amputation, *TA* traditional amputation, *TT* transtibial amputation, *TF* transfemoral amputation, *DH* disarticulation of the hip, *AWD* alive without disease, *DOD* died of disease

The patients included in this study had unresectable tumors with the following characteristics. First, limb salvage surgery was difficult and risky because of unplanned contaminated surgery, partial main vascular involvement, inadequate amounts of soft tissue covering, reconstruction difficulties, leg length discrepancies, etc. Second, the tumor did not invade the proximal epiphysis, and the femoral neck, so the intertrochanter of the femur or the tibial tubercle could be preserved. Patients were excluded from this study when, first, there were multiple metastatic diseases; second, the tumor affected the acetabulum or the lymph nodes; third, safety margins could not be achieved even after amputation.

Standard neoadjuvant chemotherapy was performed when the pathological diagnosis was confirmed as sarcoma. The chemotherapy protocol for osteosarcoma involved methotrexate (MTX, 10-12 g/m2), Adriamycin (ADM, 60 mg/m2), cisplatin (DDP, 100 mg/m2), and ifosfamide (IFO, 2.5 g/m2/day). The intensified “AVI” protocol, which included ADM (60 mg/m2), vincristine (1.5 mg/m2), and IFO (2.5 g/m2/day), was given to patients with Ewing’s sarcoma every 14 days for a total of 6 cycles preoperatively and 12 cycles postoperatively.

### Surgical technique

**Fig. 1:**
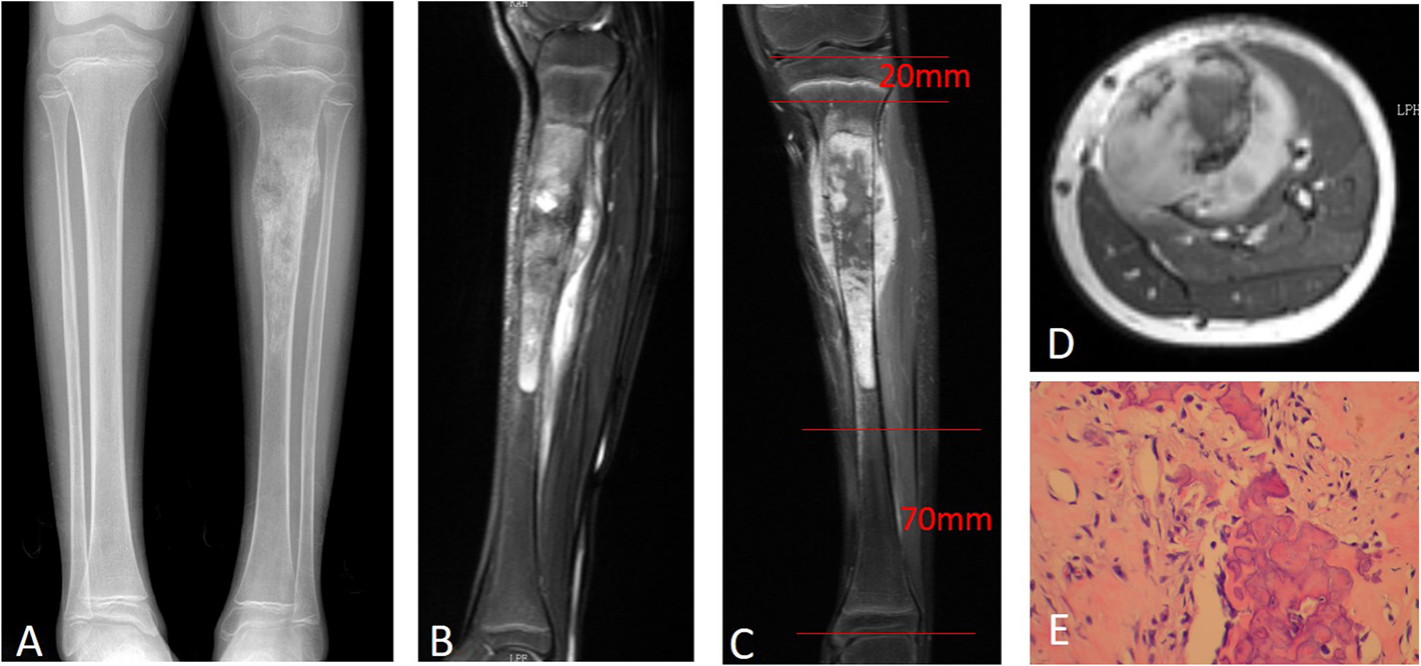
Preoperative image findings on the extent of osteosarcoma invasion in the tibia of a patient. **a** The tumor was located at the proximal tibia according to plain radiography. **b** and **c** Sagittal and coronal MRI views show that the margin of the tumor was just 2 cm below the joint surface of the knee. **d** The MRI cross-sectional view indicated that the posterior tibial vessels could be preserved. **e** HE staining of the tumor tissues

**Fig. 2:**
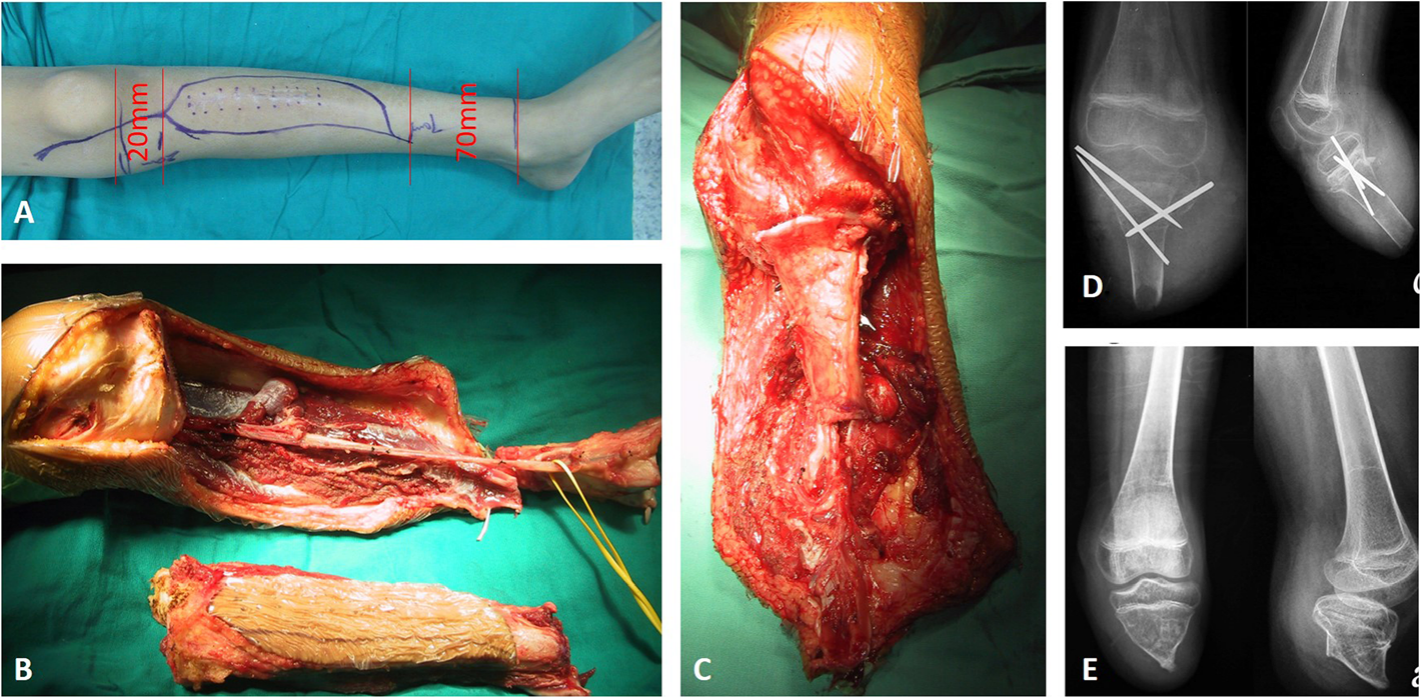
Radical removal of the tumor and preservation of the blood supply on the distal tibia. **a** Incision was marked preoperatively. **b** Tumor and surrounding tissues were resected 2 cm below the knee. The tumor and surrounding tissues were removed. **c** Osteosynthesis with Kirschner wires and preservation of the tibial epiphysis. **d** X-ray performed 1 month after the operation. **e** X-ray performed 2 years after the operation

**Fig. 3:**
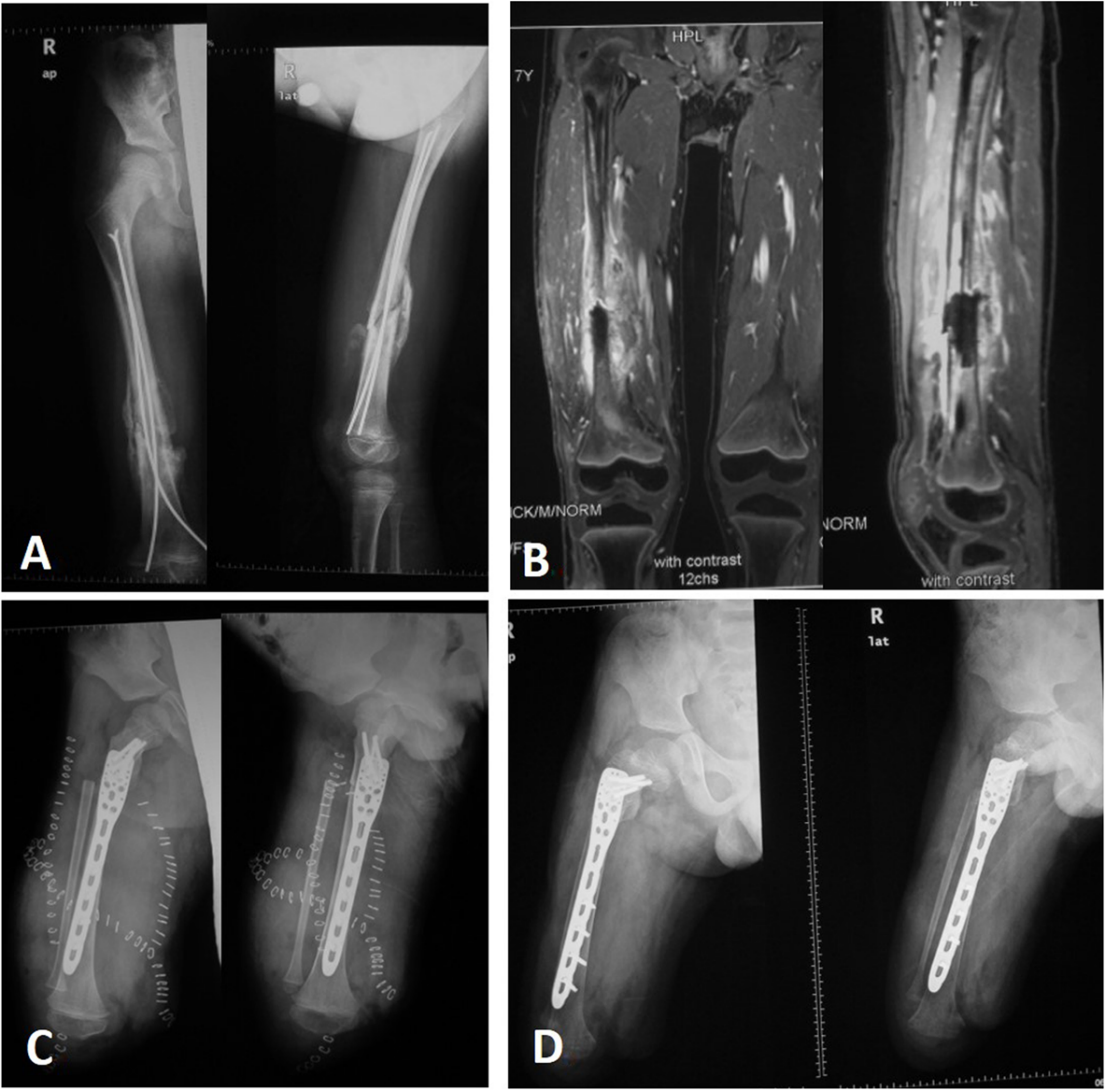
Plastic lengthening amputation of the proximal femur in a 7-year-old boy. **a** The contaminated operation was performed in the femur of a patient with osteosarcoma who was misdiagnosed with traumatic fracture and intramedullary fixation with two Steinmann pins. **b** Coronal and sagittal MRI views show that the contaminated pins reached just below the greater trochanter (5.5 cm stump left). **c** X-ray shows that the tibia was fixed to the remnant femur with an addition of 18 cm to the stump after the operation. **d** X-ray was performed 2 years after the operation

Wide resection was planned and performed in each patient according to the MRI, biopsy, and unplanned surgery results. The proximal cut was at least 2 cm from the metaphysis in the proximally involved bone. The distal cut was made with a margin of at least 3 cm (Fig. 1). If the tumor involved the main vasculature, the main vasculature was resected, and vascular anastomosis was performed with the distal posterior tibial artery and vein. In detail, for the sarcoma of the tibia, if the posterior tibial vessels could be exposed with a clear margin, vascular anastomosis was not necessary; however, if the posterior tibial vessels involved the sarcoma, vascular anastomosis was performed between the proximal end of the posterior tibial vessel and the distal end of the posterior tibial vessel. The nutrient vessels of the tibia were preserved carefully. The tumor-affected part of the tibia was resected with a safety margin, and the tibial tubercle was preserved to maintain the attachment of the patellar tendon (Fig. 2a-c). The ankle joint was removed. The residual tumor-free part of the tibia was turned approximately 180° and osteosynthesized to the metaphysis of the proximal tibia by using crossed Kirschner wire fixation (Fig. 2d). The gastrocnemius muscle was transferred anteriorly for suturing with the deep fascia and periosteum in order to cover the distal reunion bone. Paired control patients underwent traditional amputation via a transfemoral approach. For the patients with femoral sarcoma, a similar procedure was performed. The diaphysis and distal femur were widely resected with all soft tissue and skin with or without vessels. The residual proximal femur with the femoral neck and intertrochanter was preserved. Reunion of the vascularized distal tibia was performed with the remnant femur with the use of a reconstruction plate (Fig. 3). If all the vessels of the lower extremity were exposed with a clear margin, vascular anastomosis was not necessary; however, if any parts of the vessels involved the sarcoma, vascular anastomosis was performed between the proximal end of the perforating vessels and the distal end of the tibial vessels.

Vascular anastomoses were performed between the distal ends of the posterior tibial vessels and the branches of the perforating or proximal anterior tibial vessels. According to the surgical plan, 2 or 3 proximal branches that had been well marked and protected were chosen for the anastomosis. Similar diameters and sufficient lengths of the proximal vessels were recommended. The whole tumor and peritumoral tissues were taken H&E staining and IHC to confirm the type of tumor and resecting boundary.

Chemotherapy was resumed immediately after the stitches were removed, and the patients initiated passive or active exercises. The Kirschner wire was extracted 3 months later if bone healing was detected on X-ray. Prosthetic fitting was carried out 3 to 6 weeks after the operation, and walking exercises were then initiated.

### Follow up

Patients were followed up every 3 months for the first and second years, every 4 months for the third year, every 6 months for years 4 and 5, and yearly thereafter. For every follow-up, patient history was taken, and physical examination, X-ray imaging of the lesion site, and CT scan of the lungs were performed; PET-CT was performed if potential metastasis or recurrence was noticed.

### Functional evaluation

The functional outcomes were described and graded quantitatively according to the most comprehensive functional rating system of the locomotor index [10]. This functional rating system can be extended to cover more active amputees by considering its “advanced activities” subscale separately. Functional data were available for all patients at the 12-month follow-up. In addition, the patient’s functional outcome of the artificial limb was evaluated using the musculoskeletal tumor society score (MSTS), which consists of six components for the lower limb: pain, function, emotional acceptance, need for walking aids, walking, and gait. The best score for each item is 5 (range from 0-5). The values of each of the six components are added [11].

### Statistical analysis

The Kolmogorov-Smirnov (K-S) normality test was performed on the differences between PLA and TA each data set with significance established at *P* = 0.05. Paired *t* tests were used to compare the differences in the locomotor index functional score and MSTS score between the plastic lengthening amputation group and the traditional amputation group. *P* values were two-sided, and a *P* value of < 0.05 was considered statistically significant. All statistical analyses were carried out by using SPSS version 13.0 (SPSS Inc. Chicago, IL, USA).

## Results

### Baseline characteristics of patients

**Fig. 4:**
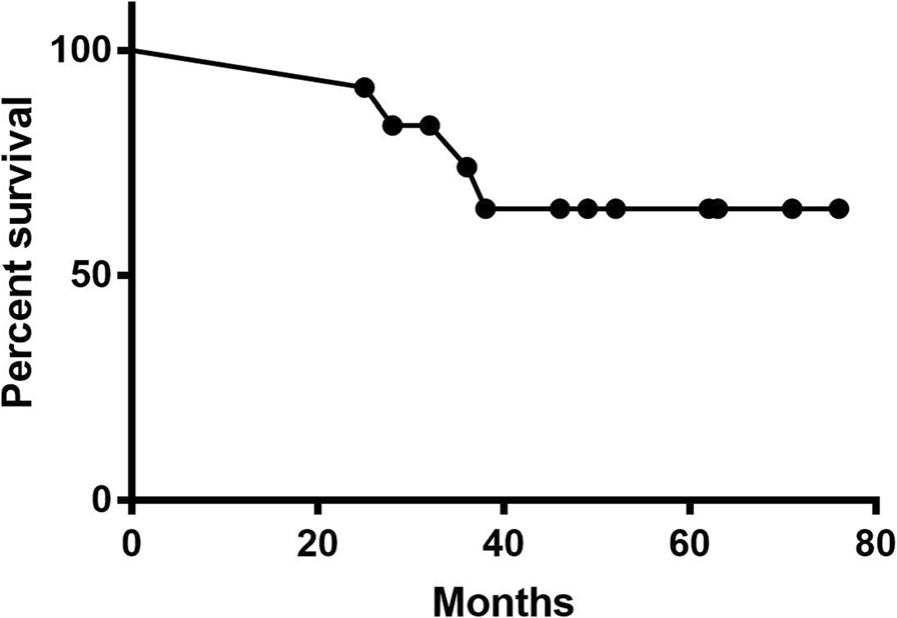
The overall survival in the twelve patients. Three patients (2 PLA group and 1 TA group) experienced pulmonary metastasis and died. Average follow-up 48.17 months (range 25 to 76)

Six pairs of patients matched for age, sex, location, pathological type, and final prosthesis underwent individually designed plastic lengthening amputation with vascularized autografts or traditional amputation between January 2005 and December 2007. Four patients were male, and eight patients were female. The average age was 9 years old (SD = 2.38; range 6 to 13 years old). All of the patients underwent neoadjuvant and adjuvant chemotherapy following the consensus of our interdisciplinary tumor board. The overall survival was shown in Fig. 4, the average follow-up period was 48.17 months (SD = 9.46; range 25 to 76 months). No local recurrence was detected after surgery, but three (25%) patients (two patients who underwent PLA and one patient who underwent TA surgery) had pulmonary metastasis in the second year and died in the third year post-operation.

Three of these patients underwent vascular anastomosis between the proximal end of the perforating or posterior tibial vessels and the distal end of the posterior tibial vessels because a portion of the vessels involved sarcoma. The average length of the remnant tibia after tumor resection was 2.4 cm (SD = 0.54; range 2.0 to 3.6 cm). The average added length of the free tibia after plastic lengthening amputation was 5.2 cm (SD = 1.13; range 4.0 to 6.2 cm). The average length of the preserved femur was 6.1 cm (SD = 0.91; range 5.2 to 7.0 cm). The average added length of the femur after plastic lengthening amputation was 15.8 cm (SD = 3.66; range 10.5 to 18.6 cm). Vascularized bone grafts and the remnant bone were fused in an average of 3.3 months (SD = 0.46; range 2.5 to 4.5 months) according to X-ray. Remodeling of the remnant shaft was observed to fit the artificial limb during the follow-up.

### Functional outcomes

**Fig. 5:**
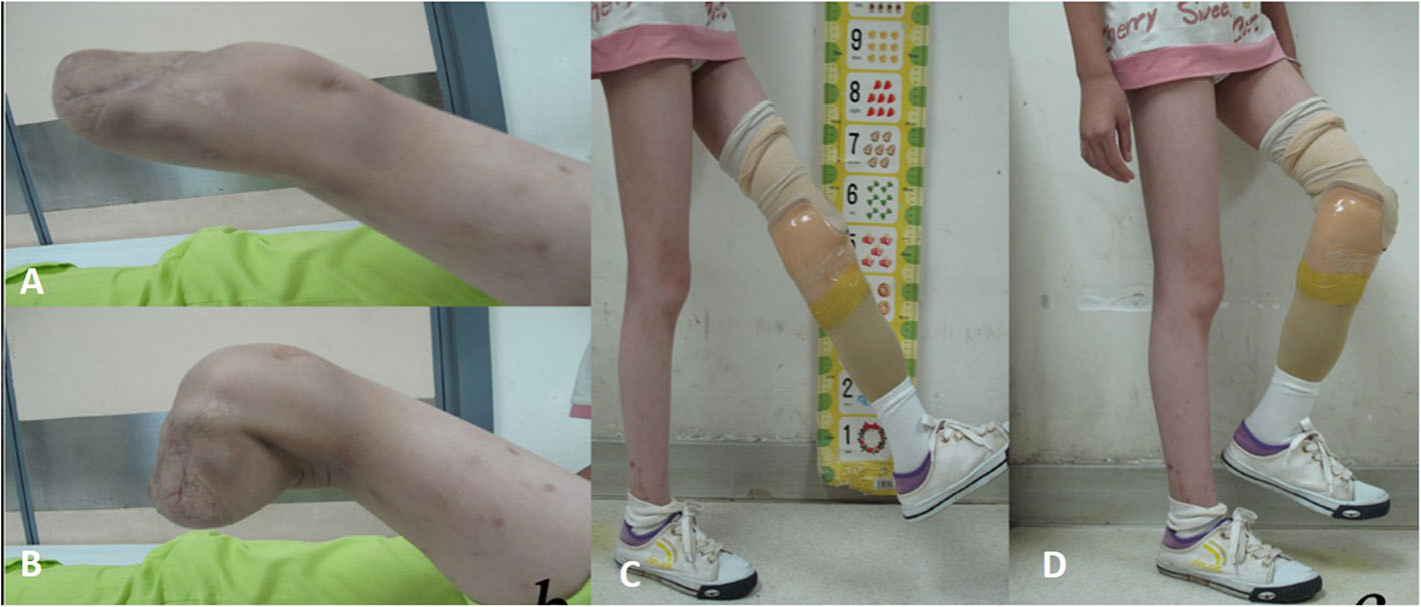
The functional outcome of one PLA case after 3 years of follow-up. **a** and **b** Bone union and a remnant stump 6 cm in size were observed. **c** and **d** The preserved joint has satisfactory extension and flexion function with or without the artificial limb

After the operation, patients with plastic lengthening amputation were able to achieve a satisfactory gait when walking and were able to squat after using the prosthetic without the aid of crutches (Fig. 5). The average locomotor index of the affected limbs was 32.67 (SD = 5.89; range 24 to 39) in the plastic lengthening amputation group, but that was 19.50 (SD = 7.87; range 10 to 25) in the traditional amputation group. The locomotor index was statistically significant between the plastic lengthening amputation group and the traditional amputation group (paired *t* test *t* = 10.771, *P* < 0.0001).

**Fig. 6:**
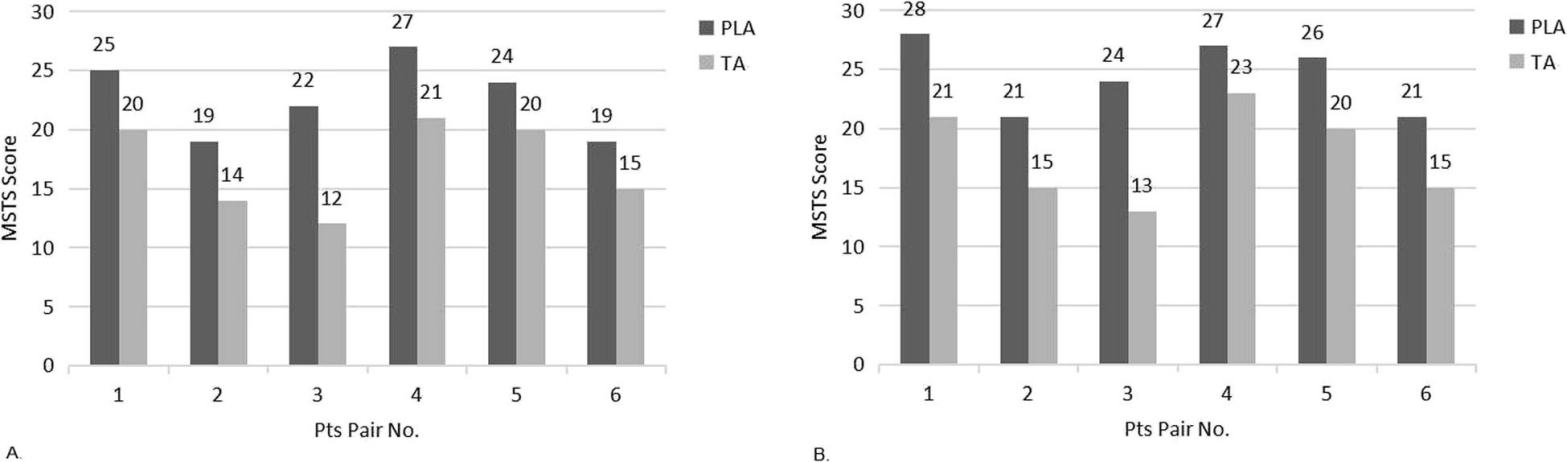
The MSTS score analysis of functional outcome in the two groups at 6 months (**a**) and 12 months (**b**) postoperation

Functional evaluations were also performed with the Musculoskeletal tumor society (MSTS) scoring system after routine physiotherapy and follow-up. Functional scores were measured at 6 and 12 months postoperatively (Fig. 6). Among the 6 patients with PLA, the functional scores were 22.67 ± 1.33 and 24.17 ± 1.45 at 6 and 12 months, respectively. However, in the 6 patients with TA, the functional scores were 17.00 ± 1.549 and 17.83 ± 1.64 at 6 and 12 months, respectively. The mean functional scores of the PLA group were significantly higher than that of the TA group at different follow-up times (*P* < 0.05). These results indicated that the patients would have better functional outcomes with the treatment of plastic lengthening amputation by vascularized bone grafts than with traditional amputation.

### Complications

One patient experienced wound complication after tibial plastic lengthening amputation. Two patients with PLA treatment-experienced bone resorption because of a lack of blood supply and disuse atrophy at 2 years after surgery.

## Discussion

Approximately 75% of osteosarcoma occurs around the knee joint. The application of neoadjuvant chemotherapy reduces the risk of relapse and metastasis [2, 3, 7]. However, in some conditions, limb salvage surgery could be too risky to perform, such as in cases of large masses involving mostly soft tissue and even partially involving main vascular tissue, in cases of unplanned contaminated surgery, and in other conditions that do not allow for limb salvage. Amputation is still the safe choice for this minority of patients. Rotationplasty, a reconstruction method used to restore the function of the knee joint with the ankle joint after the tumor-affected knee is resected, has been proven to be a mature operative technique that allows for good function in patients at follow-up [8]. However, the disadvantages of this method including the technical complexity and the long duration and unfavorable cosmetic appearance of the rotated leg with the toes pointing backward were hardly accepted by both patients and their parents [3, 5, 7].

For some patients, it is sometimes necessary to amputate at a level very close to the joint to widely remove the tumor. Whether to keep the short stump is challenging because it may not be possible to apply the prosthesis successfully to the short stump and to attain function in the adjacent joint. However, the function of transtibial amputation is better than that of transfemoral amputation, and the function of transfemoral amputation trumps that of hip disarticulation. Therefore, musculoskeletal oncologists wish to preserve transjoint residual limbs for as long as possible under the premise that the tumor is radically removed. The length of the stump plays an important role when using prostheses, and lengthening of the short stump appears to be the solution. There are several reports of some doctors using allografts or metal prostheses to lengthen the limb so that the function of the limb is greatly improved [12–17]. Wilkins introduced allograft lengthening as a new method at the International Society of Limb Salvage (ISOLS) conference, which was held in Boston in September 2009 [12].

In the current study, we named this kind of surgery as “plastic lengthening amputation” in reference to the domestic and international literature. Plastic lengthening amputation preserved the original anatomical structure and dynamic system, avoiding alterations of biomechanical flexion and extension of the knee and hip joints. More importantly, this procedure achieved a sufficient stump length for functional use of the prosthesis owing to the preservation of the distal femur and distal tibia and sometimes even the epiphysis of the proximal tibia. Furthermore, the force moment greatly increased with leg length; thus, the function of the affected limb is greatly improved. Compared with stump lengthening with allografts [18], endoprostheses, and other bone lengthening techniques, plastic lengthening achieved a shortened operative time, an excellent postoperative recovery, an easy technique, simple prosthesis assembly, and a lower cost in indicated patients.

There were strict criteria that patients must fulfill in order to be eligible for plastic lengthening amputation. We mainly considered adopting a prosthesis when the tumor was extensive and when there was no vascular or nervous compromise. Based on our experience, we regard the following as indications for plastic lengthening amputation: first, no systemic metastasis is found, or lung metastasis is present but can be controlled by surgery and/or chemotherapy. Second, if sarcoma is present in the proximal tibia, the patellar tendon junction and epiphysis can be preserved after wide resection with a safe margin. Third, the posterior tibial vessels are not compromised or vascular anastomosis can be performed safely, at least an ideal marginal resection can be achieved, and the local recurrence is not higher than that of limb salvage surgery. Similar indications were applicable in cases of proximal femur sarcoma. Our goal was to transfer hip disarticulation to above-knee amputation, thus lengthening the residual bone and improving function. Based on our research, patients who underwent plastic lengthening amputation had a better functional outcome and were satisfied compared with those who underwent traditional amputation with an artificial limb.

There were some notions for successful plastic lengthening amputation. First, the distal-proximal blood supply is mainly provided by intramedullary feeder vessels, which might be damaged by distal turmeric bone resection. Therefore, surgeons should avoid stripping the periosteum of the distal autograft so that the blood supply from the periosteum is not compromised. Second, the vessels should not be stretched excessively while dividing them so that the contracture of blood vessels can be avoided. During the operation, we can judge the blood supply of the autograft by observing whether there is an effusion of blood in both ends. Third, the autograft is turned upside down, and the transverse section close to the ankle joint is fused with the sectioned plane below the knee so that the contact area is increased. This procedure not only increases stability but also improves the blood supply of the autograft. In addition, transferring the gastrocnemius myofascial flap over the ends of the bones might lead to increased torque while flexing the knee. Flexion deformities may appear after surgery. To solve this problem, we encouraged patients to perform extensive quadriceps-contraction training or to undergo a second surgery to release the starting point of the gastrocnemius.

In our series, wound healing problems occurred in one patient due to local hypertension caused by exudation. Other than that, there were no major complications such as infection or non-healing. However, our sample size was small; as was known, this procedure was indicated for selected sarcoma cases where routine limb salvage was deemed too risky. A more accurate complication rate could be determined with more patients and longer follow-up times. Hence, we hope that PLA is adopted and applied by more musculoskeletal oncologists to improve the procedure furtherly.

Although plastic lengthening amputation had strict inclusion criteria and was rarely indicated, it had the advantages of preserving the knee joint structure and function, transforming hip disarticulation to transfemoral amputation, preserving the growth of the epiphysis, and allowing for fast healing of the vascularized autograft. Therefore, the function of the limb after prosthetic fitting fixation was greatly improved. Other advantages included the shortened surgical duration, easily handled technique, quick recovery, and accessibility of prosthetic fixation.

## Conclusion

In summary, plastic lengthening amputation after excision of malignant lesions is a modified surgery for above-knee and below-knee amputations. This procedure is a valuable addition to the procedures available to tumor surgeons and, in selected patients, is superior to traditional amputation.

## Data Availability

The data that support the findings of this study are available on request from the corresponding author. The data are not publicly available due to privacy or ethical restrictions.

## Abbreviations

PLA: Plastic lengthening amputation
TA: Traditional amputation
MSTS: Musculoskeletal tumor society score
MTX: Methotrexate
ADM: Adriamycin
DDP: Cisplatin
IFO: Ifosfamide
ISOLS: International Society of Limb Salvage
TT: Transtibial amputation
TF: Transfemoral amputation
DH: Disarticulation of the hip
AWD: Alive without disease
DOD: Died of disease
